# The Evolution of Web-based Medical Information on Sore Throat: a Longitudinal Study

**DOI:** 10.2196/jmir.5.2.e10

**Published:** 2003-06-13

**Authors:** Vincenzo Currò, Paola Sabrina Buonuomo, Paola De Rose, Roberta Onesimo, Andrea Vituzzi, Alessandro D'Atri

**Affiliations:** ^1^Università Cattolica del Sacro CuoreIstituto di Clinica PediatricaRomeItaly; ^2^LUISS "Guido Carli"Centro di Ricerca sui Sistemi InformativiRomeItaly

**Keywords:** World Wide Web, Internet, evolution, sore throat, quality of contents, quality control, longitudinal studies

## Abstract

**Background:**

The content of a page can change and is likely to change over time; this is one of the useful qualities of the Web, but also a dangerous one.

**Objective:**

To monitor the evolution of Web page contents on sore throat over a 3 year period.

**Methods:**

Two medical doctors independently evaluated 34 Web pages on sore throat. Pages were found using a metasearch engine. The evaluation factors were: the adherence of medical contents to a gold standard (American Academy of Pediatrics recommendations) composed of 5 subfactors (epidemiological, clinical, complications, diagnosis, and therapy); the completeness of the contents in terms of considered/missed factors of the gold standard; references to medical literature; and a specified last update of the page. During the observation period these sites were revisited twice, after 28 and 39 months, to examine any changes therein since the first visit.

**Results:**

The degree of adherence to the gold standard did not significantly change. Variations (both positive and negative) were recorded solely with regard to the update and references factors as well as with regard to the availability of the pages over time (18% disappeared during the observation period).

**Conclusions:**

In 3 years medical contents have not changed significantly and despite the contemporary epochal Internet revolution (in terms of, eg, technology, graphics, and access) and the increase in the number of sites dealing with the issue of sore throat, there has been no corresponding qualitative increase in the contents of the pages monitored.

## Introduction

The Internet is a relatively-recent phenomenon and its evolution and use by nonprofessionals have developed especially in the last 5 years. The usefulness of medical cyberspace continues to grow and links, search engines, and the presentation of information as well as users' Web-surfing skills all seem to be steadily improving. The use of the Internet is also rapidly increasing in Italian households. Our previous study carried out on a population of parents in 1999 showed that approximately 19% of an unselected sample of parents used the Internet to acquire medical information [1]. The ability to obtain medical information quickly, cheaply, and in the home represents an important development for better-informed participation in the care of children. From the point of view of pediatricians the dissemination of correct medical information to parents is an essential aspect of childcare. In our evaluation we did not consider such factors as usability and aesthetics. These areas, which are most certainly of interest in parents' consideration of a good Web page, are discussed in another study now in progress. Despite growing interest in evaluating health information on the Web, professional users say information quality is a problem [2- 6]. Eysenbach [7], in a recent systematic review [7] on what he called "infodemiology" [8] studies, i.e. studies where investigators evaluated the quality of health information on the web, reported that the greater part of studies (70%) concluded that quality is poor on the Web, while only 9% gave a positive statement.

The content of a page can change and is likely to change over time; this is one of the useful qualities of the Web, but also a dangerous one. Although many attempts have been made to assess, control, and assure the quality of Web-based medical information [9- 11], there is no single standard strategy for evaluating Web pages available to nonprofessional users such as parents. The Web is truly an international press and is essentially unregulated [12]. Although the cited authors provide relevant results, their analysis is on the status of the quality and not on its evolution over time. Since the Internet is continuously changing, our evaluation during a 3-year period could be an additional element for investigating this phenomenon. A contribution in this direction is offered by the study of Pandolfini et al [13] who replaced their study of 1997 [4] and reevaluated the quality of the same sites included in that study, as well as that of a more-recent sample of pages, using the same methodology. Li et al [14] conducted a prospective systematic review of Web sites related to back pain, using 5 search engines, during a 2-year period. In our study we carried out an evaluation of the content of medical Web sites directed to parents and we repeated the study of the same Web pages after a 24 and a 39 month period respectively.

## Methods

On August 12 1998 we searched the World Wide Web for the first time using Metacrawler, a metasearch system that in 1998 combined 6 different search engines (Alta Vista, Excite, Infoseek, Lycos, Webcrawler, and Yahoo). Because of the differences among the search engines (eg, Infoseek is a random-search tool and Alta Vista ranks the requested references in order of relevance) a metasearcher combines such different approaches in a unique "independent" collection that could be linked to the way in which parents — who have variable experience, culture, and computer ability — perform the search.

We used the English words: *sore throat* and *pharyngitis* and the corresponding Italian translations: *mal di gola* and *faringite*(ie, the medical condition and its symptom, because the parents frequently use the same words for their research) without Boolean operators, as Metacrawler did not use them in the search; the results displayed 97 Web sites. As occurs in all Internet searches, some inappropriate documents appeared. We excluded all pages in which the words of our search appeared in a nonmedical context; ie, we excluded all pages created by commercial ventures and corporate sites sustained by advertising and sales of commercial products (eg, candies, oral spray, and herbal tea) as well as pages related to veterinary purposes. After the exclusion of the inappropriate sources, 34 Web sites were included in the study. These Web sites were all created by health care authorities (eg, American Medical Association and American Academy of Pediatrics), hospitals (eg, Children's Hospital of Iowa and St. James Hospital), departments of public health (eg, Hawaii, Illinois, and Bethesda), research foundations (eg, MayoClinic), or other lay health care organizations (eg, WellnessWeb, MedicineNet, HealthyNet). The list of evaluated sites is in Table 1.

To evaluate the quality of each Web site, we considered the adherence to some of the criteria suggested in literature available in 1998 on this topic: suitability of the medical contents, scientific citations, and date of creation and/or of the last update. We compared the medical contents of all these Web sites with the recommendations of the Committee on Infectious Diseases of the American Academy of Pediatrics [15], whose guidelines did not change between then and the last edition published in 2000 [16].

**Score Criteria**

Adherence

The median score of the present factors was used as the global evaluation of *adherence*. Since our gold standard consists of 5 factors (epidemiological, clinical, complications, diagnosis, and therapy), each factor was evaluated according to the following scale: 1 (low, in case of errors or no adherence); 2 (medium-low adherence); 3 (medium adherence); 4 (medium-high adherence); and 5 (full adherence).

Completeness

*Completeness* was determined according to the number of considered/missed factors of the gold standard: 1 (the worst, in case of 1 factor), 2 (bad, 2 factors), 3 (medium, 3 factors), 4 (good, 4 factors), 5 (the best, all factors).

References

*References*: 1 if the page listed some pertinent references about its contents.

Update

*Update*: 1 if the page contained the date of creation (or its last update).

According to these criteria, the quality of the Web pages was independently evaluated by 2 medical doctors (VC, a senior pediatrician, and PSB, a resident) to validate the medical information for parents. Each doctor evaluated every Web site individually without knowing the site's address or author. When the 2 authors disagreed, to ensure the reliability of the rating they talked about the disagreement until they reached a common value.

On December 12, 2000 and on November 12, 2001 all 34 sites included in the study were revisited to see if the pages still existed or if they had been substituted with new pages. All pages were reevaluated with the same methodology, comparing the content with copies of the original pages. The missing pages were manually searched in the Web (by means of Altavista, Google, and other search engines) starting from the address and/or the contents of the previously-evaluated pages. The results were analyzed through Wilcoxon's paired signed rank test and the McNemar test.

**Table 1:** Sites evaluated and their respective homepage URL

**Organization**	**Homepage URL**
Abersychan Surgery	http://www.abersychan.demon.co.uk/
American Academy of Pediatrics	http://www.aap.org/
American Medical Association	http://www.ama-assn.org/
Association of State and Territorial Directors of Health Promotion and Public Health Education (ASTDHPPHE)	http://www.astdhpphe.org/
Baltimore County Public Schools	http://www.bcps.org/
Beijing Scene Publishing	http://www.beijingscene.com/
National Center for Emergency Medicine Informatics	http://www.ncemi.org/
Children's Medical Center of Dallas	http://www.childrens.com/
ComWeb	http://espaceweb.qc.ca/
Darthmouth-Hitchcock	http://www.healthimprov.org/
Drugbase	http://www.drugbase.co.za/
Eastern Wyoming College	http://ewcweb.ewc.whecn.edu/
http://www.fcr.re.it/	http://www.saninforma.it/
Group Health Cooperative	http://www.ghc.org/web/
Hawaii Department of Health	http://mano.icsd.hawaii.gov/doh/
HealthWorld Online	http://www.healthy.net/
HealthAnswers,Inc	http://www.healthanswers.com/
Housecall Medical Resources, Inc.	http://www.housecall.com/
linois Department of Public Health	http://www.idph.state.il.us/
Kenyon College	http://www.kenyon.edu/
MayoClinic.com	http://www.mayoclinic.com/
MedicineNet, Inc.	http://www.medicinenet.com/
Medscape Portals, Inc.	http://www.medscape.com/
National Institute of Allergy and Infectious Diseases	http://www.niaid.nih.gov/
Nightime Pediatrics Clinics, Inc.	http://www.nightimepeds.com/
Seton Hall University	http://studentaffairs.shu.edu/
St. James's Hospital	http://www.stjames.ie/
The Nemours Foundation	http://www.kidshealth.org/
University of Iowa	http://www.medicine.uiowa.edu/
University of Colorado at Boulder	http://www.colorado.edu/
University of Iowa Health Care	http://www.uihealthcare.com/
University of Missouri-Rolla	http://www.umr.edu/
University of Rochester	http://www.rochester.edu/
WellnessWeb	http://www.wellnessweb.com/

## Results

During the observation period, 6 (18%) of the 34 pages monitored disappeared between August 1998 and November 2001; 3 pages were no longer available after 28 months with the remaining ones no longer active by 39 months. Table 2 summarizes the results of our survey for 1998, 2000, and 2001 and shows for each variable (adherence, completeness, update, and references) the number of Web sites for each score.

**Table 2:** Comparison of scores for 1998, 2000, and 2001

**Score Criteriaand Score**	**Number of Web Sites**		
	**Year**		
	**1998**	**2000**	**2001**
Adherence			
1	3	3	2
2	1	1	1
3	5	4	4
4	5	5	3
5	20	18	18
Completeness			
1	0	0	0
2	4	4	4
3	6	4	3
4	8	6	6
5	16	17	15
References			
0	29	27	24
1	5	4	4
Update			
0	22	19	16
1	12	12	12
Number of sites evaluated	34	31	28

Because only 1 Web site showed a large degree of variation in terms of adherence and completeness to the gold standard, no statistical analyses were performed. Most variations relate exclusively to update and references; to evaluate their evolution, we made the Wilcoxon's paired signed rank test and the McNemar test for the pairs of years 1998-2000 and 1998-2001. The differences were not significant ( *P*> .05) for update and references for either pair of years. The pair 2000-2001 was not considered because there were not differences between respective values, the only difference was in the number of available sites.

## Discussion

If it is true that the Internet is continuously developing as regards the quantity of information available and the number of sites online, it seems that the same cannot be said for the quality of the information provided. This leads one to have doubts as to the effective greater dynamism of Web publications compared to publications printed on paper. Only 3 sites in our sample modified the pages relative to sore throat during the observation period and in 2 cases the changes did not have any significant impact on quality. With the exception of 1 site, the medical contents of the reevaluated sites remained similar to the initial version; apart from the foregoing, improvements were recorded only in aesthetics (2 sites) and a request for HON certification (1 site).

Of the 5 gold standard factors, the least common one is *complication*(about 60% of the sites) whereas *clinical* is in all sites. The factor that adheres least of all to the gold standard is *therapy*(72%). Overall average adherence is 81%. Only 12.5% of the sites give any indication of references. Only 33% of the sites specify the date of creation or of the last update. In our opinion it is very important for the reader to know the date the page was written; a recent date would imply possibly-better information, especially for the sites with a bad evaluation. In addition, some authors [7- 18,9] included the date of creation or update of the page among the items to consider when you want to evaluate health-related Web sites. In particular Abbott [17] included the update under the category of "content." Although this article was published after the beginning of our study, its conclusions reinforced our considerations. The low number of sites that dedicate space to complications is probably because these sites are addressed to more "impressionable" nonprofessionals (often parents) whose anxiety for their children's health dictates that it is better that matters like this are discussed personally and directly with a pediatrician. Of greater concern is the poor quality of therapeutic advice, since dealing with the issue inadequately means there is a risk of people treating themselves erroneously.

In the observation period the relevant changes were restricted to *update*(15 sites) and *references*(6 sites). An unexpected result was that the number of improved sites was the same as the number of those that became worse; hence the overall average result was the same as before. This leads one to wonder if such a trend is limited to the pages on sore throat or whether it is also applicable to other contexts.

It was not always trivial to find a missing page through the initial address or via a simple path starting from the home page. The Web administrator often made a major change in the location of the page in the site. In some cases there were no direct links to reach the new location; instead, the new page was only accessible by using a search engine In 1 case, the Web site on which the page was published ceased to exist.

Our research was limited in terms of the observation period, the number of monitored sites and the unique medical issue considered. The idea of conducting a broader and more-systematic analysis of the evolution over time of the pediatric information available to parents via a suitable "Internet observatory" is an important challenge. Unfortunately, the huge number of sites and issues to be monitored require a correspondingly huge amount of resources; nevertheless there is the need to continuously improve the efforts to provide the final user such services.
